# Microfiltration of glass ionomer restorations in teeth treated with silver diamine fluoride: A scoping review

**DOI:** 10.4317/jced.62928

**Published:** 2025-07-01

**Authors:** Ana Lucia Avellaneda-Gonzales, Maria Elizabeth Cruz-Flores, Rosa Josefina Roncal-Espinoza, Alfredo Carlos Manuel Rendon-Alvarado

**Affiliations:** 1 Student, School of Dentistry, Universidad Católica Santo Toribio de Mogrovejo, Chiclayo, Peru; 2 Master in Stomatology. Specialist in Pediatric Dentistry. Undergraduate and Postgraduate Professor, School of Dentistry, Universidad Católica Santo Toribio de Mogrovejo, Chiclayo, Peru; 3 Master in Stomatology. Specialist in Restorative and Esthetic Dentistry. Undergraduate and Postgraduate Professor, School of Dentistry, Universidad Católica Santo Toribio de Mogrovejo, Chiclayo, Peru; 4 Master in Stomatology with a Mention in Oral Rehabilitation. Specialist in Oral Rehabilitation. Undergraduate Professor, School of Dentistry, Universidad Católica Santo Toribio de Mogrovejo, Chiclayo, Peru

## Abstract

**Background:**

The current approach to dental caries focuses on early diagnosis and intervention at initial stages. In the presence of a carious lesion, a minimally invasive strategy is favored. Within this context, materials have been developed to promote remineralization, prevention, and conservative treatments, with silver diamine fluoride (SDF) standing out as an effective alternative. The purpose of this scoping review is to evaluate, through a literature review, the success of restorations in teeth treated with SDF, using microleakage as the evaluation parameter.

**Material and Methods:**

A search was conducted in electronic databases including PubMed, Virtual Health Library, EBSCOHost, SCOPUS, ProQuest, and ScienceDirect. The search was carried out up to May 24, 2024, following the PRISMA (Preferred Reporting Items for Systematic Reviews and Meta-Analyses) guidelines.

**Results:**

A total of 298 articles were retrieved, of which only 8 met the inclusion criteria and were used for the literature review. The selected studies underwent a quality control review to assess the success of restorations in teeth treated with SDF.

**Conclusions:**

Although information on the effect of SDF on the adhesion of resin-modified glass ionomer restorations is limited, some studies show no correlation indicating that prior SDF treatment causes microleakage in such restorations.

** Key words:**Dental caries, Preventive dentistry, Dental restoration, Silver diamine fluoride.

## Introduction

The current management of dental caries is based on early diagnosis and intervention during the initial stages, focusing on the treatment of incipient non-cavitated lesions or early dentinal lesions on smooth surfaces. Once a carious lesion is present, the preferred approach is to treat it with minimal intervention, aiming to preserve as much tooth structure as possible when replacing restorations. Therefore, materials that support remineralization, prevention, and minimally invasive treatment have been sought, with silver diamine fluoride (SDF) emerging as a viable alternative (1,2).

SDF is typically used at a 38% concentration. It is a topical, painless, and cost-effective agent employed in many countries to arrest dental caries. SDF exhibits antimicrobial properties while simultaneously protecting the teeth by halting and preventing further decay. When applied to enamel and dentin, an interaction occurs between phosphate and calcium ions, leading to the formation of low-solubility fluorohydroxyapatite—a key factor in preventing or reducing dental caries. Additionally, one of the main advantages of SDF is that carious tissue does not need to be removed prior to its application, simplifying clinical procedures (3-5).

However, due to its silver content, SDF presents adverse effects, such as the dark staining of carious lesions. In addition, when the cavity remains open, it can become a site for plaque accumulation, which may lead to reactivation of the carious process. For this reason, clinicians often restore teeth treated with SDF to improve patients’ oral hygiene (2,3-6).

Dental restorations can be performed using various materials, such as composite resin and glass ionomer cement—a material commonly used in the atraumatic restorative treatment approach—resin-modified glass ionomer, and polyacid-modified composite resins. Among these, the most frequently used are composite resin and resin-modified glass ionomer (5).

Some authors have reported that SDF may compromise the integrity of the adhesive bond. This is because SDF penetrates dentinal tubules, increasing dentin’s resistance to demineralization, which could interfere with the adhesion of restorative materials (3,6-11). Moreover, several studies recommend the application of specific materials following the use of SDF (4-6). One such material is potassium iodide, which can help reduce the staining caused by SDF. Another approach is the application of pit and fissure sealants over the treated area (1).

Based on the foregoing, the purpose of this scoping review is to evaluate, through a literature review, the success of restorations in teeth treated with SDF, using microleakage as the evaluation parameter

## Material and Methods

The PICO model was used, establishing the following components: *P* (Population): Teeth treated with SDF; I (Intervention): Restoration with resin-modified glass ionomer; O (Outcome): Microleakage at the adhesive interface.

- Search Strategy

The search strategy was conducted up to May 24, 2024, following the PRISMA (Preferred Reporting Items for Systematic Reviews and Meta-Analyses) guidelines. A literature review was performed in databases such as PubMed, Virtual Health Library (VHL), Scopus, ScienceDirect, ProQuest, and EBSCOhost. No time restrictions were applied to the article search.

Both MeSH terms and non-MeSH terms were used. The MeSH terms included: “silver diamine fluoride,” “diamine silver fluoride,” “dental leakage,” and “dental restoration failure.” The non-MeSH terms included: “resin modified glass ionomer cement,” “resin-modified glass ionomer,” “glass ionomer filling,” “microleakage,” and “shear bond strength.” These terms were combined using the Boolean operators OR and AND, yielding different search expressions depending on the database used.

Included articles were those published in English that focused on the topic of interest, including *in vitro* laboratory studies and experimental studies involving human populations (comparative clinical trials [CCTs], randomized controlled trials [RCTs], and non-randomized trials). Exclusion criteria comprised case reports, animal studies, book chapters, letters to the editor, literature reviews, systematic reviews, theses, and procedural manuals.

- Study Selection and Eligibility

Two researchers (A.L.A.G and M.E.C.F.) carried out the study selection process. The Rayyan tool was used to identify and remove duplicate records. Titles and abstracts were then screened according to the PICO question, followed by the retrieval of relevant full-text articles. Disagreements were resolved by a third reviewer (R.J.R.E.).

For data extraction, a spreadsheet (Microsoft Excel™, Microsoft Corporation) was used, which included the following information: year of publication, article title, author, country, study objectives, study design, population, sample, sample characteristics, sample selection, intervention performed (study groups and materials used), results (observation period, type of evaluation), and conclusions.

## Results

A comprehensive search was conducted, yielding a total of 298 articles: 8 from PubMed, 183 from Scopus, 8 from BVS, 34 from ScienceDirect, 62 from ProQuest, and 3 from EBSCOhost. During the screening process, duplicate records were removed using the Rayyan tool. A third reviewer intervened in cases of disagreement, resulting in a total of 215 articles. Titles and abstracts were then reviewed, followed by full-text analysis. Ultimately, 8 articles were included in the review, as shown in  Table 1.

- Description of the Studies

A thorough review of the selected articles was conducted to determine their characteristics, which are summarized in  Table 2.

- According to the Equipment Used

In the reviewed studies, a universal testing machine was employed to assess the adhesive strength of the restorative material (12-19). To evaluate microleakage, some studies used a stereomicroscope (12-14,16-18), one study used a light microscope (19), while others utilized Scanning Electron Microscopy (SEM) (14,19).

- According to the Type of Failure

The studies assessed various types of failure, including adhesive failure, cohesive failure, and mixed failure. Among them, adhesive failure was the most frequently observed (12-19).

- According to the Material Used

All studies used 38% silver diamine fluoride (SDF), either alone or in combination with other materials. Carious lesions were restored with resin-modified glass ionomer in most cases; some studies used only this material, while others employed additional restorative materials to compare their effectiveness. Among the studies that used only 38% SDF to assess restoration success, some compared outcomes with materials such as resin-based composite, a new self-adhesive restorative material, and resin-modified glass ionomer (12,16).

Other studies used SDF and compared it with casein phosphopeptide–amorphous calcium phosphate (CPP-ACP), followed by restoration with resin-modified glass ionomer (13). In another study, a combination of 38% SDF and potassium iodide was applied, and the tooth was subsequently restored with resin-modified glass ionomer (14,15,18). One study evaluated restorations after the application of SDF alone and SDF combined with potassium iodide (19). There were also studies in which only SDF was used prior to restoration with resin-modified glass ionomer (17).

- According to the Type of Specimen

Most studies were conducted on permanent teeth (12-15,17,18), except for those by Elazab, Sa’ada, and Uchil (14,17,19), who conducted their research on primary molars, where a higher rate of mixed failure was observed.

## Discussion

Silver diamine fluoride (SDF) is an antibacterial compound that promotes remineralization of dental tissue affected by caries due to its fluoride and silver components (7). For this reason, it is used to arrest the progression of carious lesions (1).

The growing use of SDF as a preventive treatment highlights the importance of understanding the potential for microleakage in restorations placed on teeth previously treated with this product, particularly restorations using resin-modified glass ionomer (RMGIC). Authors such as Phani Kumar *et al*. (13) compared SDF with casein phosphopeptide–amorphous calcium phosphate (CPP-ACP), a phosphoprotein capable of releasing calcium and phosphate ions into the oral environment to promote remineralization. Their findings showed a higher incidence of adhesive failure in teeth treated with SDF compared to those treated with CPP-ACP.

Most of the studies reviewed agree that no microleakage occurs in restorations of teeth treated with SDF (12-16,18-20). Abdelsattar Khaled Ahmed *et al*. (21) support these findings, stating that pretreatment with SDF does not affect the bonding of restorations with RMGIC. However, other authors, such as Sa’ada (17), report an increase in mixed failure—indicating problems in both adhesion and cohesion—particularly in primary teeth treated exclusively with SDF.

One study analyzed the integrity of the adhesive bond by comparing SDF concentrations of 38% and 3.8%, finding that the latter showed a lower failure rate (18). This difference may be attributed to the lower silver content.

Among the disadvantages of SDF is the discoloration caused by silver penetration into dentinal tubules. To address this, compounds have been introduced to reduce the black staining effect. One such compound is potassium iodide, which helps mitigate the discoloration of the treated tooth (16,18,19). A study by Gupta *et al*. (20) further noted that combining potassium iodide with SDF not only reduces staining but also enhances the adhesive strength of RMGIC.

## Conclusions

Evidence regarding the effect of SDF on the adhesion of resin-modified glass ionomer restorations remains limited. However, existing studies indicate that there is no consistent correlation confirming that prior treatment with SDF leads to microleakage in these restorations. It has been suggested that this may be influenced by the adjunctive use of potassium iodide, a material considered more effective and capable of masking the discoloration caused by silver. Further studies are recommended to confirm these findings and to assess the long-term success of such restorations.

## Figures and Tables

**Table 1:** Search Strategy.

DATA BASE	SEARCH STRATEGY
PUBMED	(("silver diamine fluoride"[Title/Abstract] OR "diamine silver fluoride"[Title/Abstract]) AND ("Resin modified glass ionomer cement"[Title/Abstract] OR "resin-modified glass ionomer"[Title/Abstract] OR "Glass ionomer filling"[Title/Abstract])) AND ("Dental leakage"[Title/Abstract] OR "Dental restoration failure"[Title/Abstract] OR "microleakage"[Title/Abstract] OR "Shear bond strength"[Title/Abstract])
SCOPUS	( ALL ( "silver diamine fluoride" OR "diamine silver fluoride" ) ) AND ( ALL ( "Resin modified glass ionomer cement" OR "resin-modified glass ionomer" OR "Glass ionomer filling" ) ) AND ( ALL ( "Dental leakage" OR "microleakage" OR "Shear bond strength" ) )
BVS	("silver diamine fluoride" OR "diamine silver fluoride") AND ("Resin modified glass ionomer cement" OR "resin-modified glass ionomer" OR "Glass ionomer filling") AND ("Dental leakage" OR "Dental restoration failure" OR "microleakage" OR "Shear bond strength")
SCIENCE DIRECT	("silver diamine fluoride" OR "diamine silver fluoride") AND ("Resin modified glass ionomer cement" OR "resin-modified glass ionomer" OR "Glass ionomer filling") AND ("Dental leakage" OR "Dental restoration failure" OR "microleakage" OR "Shear bond strength")
PROQUEST	("silver diamine fluoride" OR "diamine silver fluoride") AND ("Resin modified glass ionomer cement" OR "resin-modified glass ionomer" OR "Glass ionomer filling") AND ("Dental leakage" OR "Dental restoration failure" OR "microleakage" OR "Shear bond strength")
EBSCO	"silver diamine fluoride" AND "resin modified glass ionomer" AND ( "microleakage" OR "shear bond strength" )
DATA BASE	SEARCH STRATEGY
PUBMED	(("silver diamine fluoride"[Title/Abstract] OR "diamine silver fluoride"[Title/Abstract]) AND ("Resin modified glass ionomer cement"[Title/Abstract] OR "resin-modified glass ionomer"[Title/Abstract] OR "Glass ionomer filling"[Title/Abstract])) AND ("Dental leakage"[Title/Abstract] OR "Dental restoration failure"[Title/Abstract] OR "microleakage"[Title/Abstract] OR "Shear bond strength"[Title/Abstract])
SCOPUS	( ALL ( "silver diamine fluoride" OR "diamine silver fluoride" ) ) AND ( ALL ( "Resin modified glass ionomer cement" OR "resin-modified glass ionomer" OR "Glass ionomer filling" ) ) AND ( ALL ( "Dental leakage" OR "microleakage" OR "Shear bond strength" ) )
BVS	("silver diamine fluoride" OR "diamine silver fluoride") AND ("Resin modified glass ionomer cement" OR "resin-modified glass ionomer" OR "Glass ionomer filling") AND ("Dental leakage" OR "Dental restoration failure" OR "microleakage" OR "Shear bond strength")
SCIENCE DIRECT	("silver diamine fluoride" OR "diamine silver fluoride") AND ("Resin modified glass ionomer cement" OR "resin-modified glass ionomer" OR "Glass ionomer filling") AND ("Dental leakage" OR "Dental restoration failure" OR "microleakage" OR "Shear bond strength")
PROQUEST	("silver diamine fluoride" OR "diamine silver fluoride") AND ("Resin modified glass ionomer cement" OR "resin-modified glass ionomer" OR "Glass ionomer filling") AND ("Dental leakage" OR "Dental restoration failure" OR "microleakage" OR "Shear bond strength")
EBSCO	"silver diamine fluoride" AND "resin modified glass ionomer" AND ( "microleakage" OR "shear bond strength" )

**Table 2:** Characteristics of the Included Studies.

Author, Country and Year	Intervention	Sample
Aldowsari et al. Saudi Arabia (2023)	G I: Samples loaded with resin base; G II: New adhesive restorative material, SureFil; G III: Resin-modified glass ionomer cement.	39 permanent molars
Phanikumar, M.S. et al. India (2023)	Group 1: Pretreatment with 38% SDF + RMGIC; Group 2: Pretreatment with CPP-ACPF + RMGIC; Group 3: No pretreatment + RMGIC (control).	30 permanent premolars
Elazab M et al. Egypt (2022)	Microtensile bond strength; Group I (intervention): 38% SDF + KI; Group II (control): No treatment, stored in deionized water for 14 days followed by RMGIC. Bond strength: Group III (intervention): 38% SDF + KI; Group IV (control): No treatment, stored in deionized water for 14 days followed by RMGIC.	60 primary molars
Ghilotti J et al. Spain (2023)	SG I: SDF/KI + GV; SG II: PA + GV; SG III: PA + SDF/KI + GV; SG IV: PAA + GV; SG V: PAA + SDF/KI + GV.	15 permanent molars
Aldosari MM et al. Saudi Arabia (2022)	Subgroup A (control samples), immediate load of restorative material on sound dentin; Subgroup B, demineralized dentin, treatment with SDF and immediate load of restorative material; Subgroup C, demineralized dentin, treatment with SDF and restorative material loaded one week later.	90 permanent premolars
Sa'ada MMA et al. Egypt (2021)	G I: Dentin surfaces treated with 38% SDF solution (Elevate Oral Care, USA) for 3 minutes using a microbrush, followed by 30 s water rinse; G II: Dentin surfaces treated with distilled water for 3 min and then rinsed for 30 s.	20 primary molars
Alshahrani Saudi Arabia (2020)	G 1: Ten samples left untreated; G 2: Exposed to polyacrylic acid conditioner; G 3: Samples treated with 38% SDF; G 4: Samples protected with 3.8% SDF; G 5: Topical application of 38% SDF plus potassium iodide; G 6: Demineralized dentin treated with photodynamic therapy.	60 molars
Uchil S et al. India (2020)	G I: 10% polyacrylic acid conditioner; G II: 38% SDF; G III: 37% phosphoric acid etchant followed by 38% SDF; G IV: 37% phosphoric acid etchant followed by 38% SDF and 10% potassium iodide solution.	36 primary molars

## Data Availability

The datasets used and/or analyzed during the current study are available from the corresponding author.
